# Dehydrogenation of Betacyanins in Heated Betalain-Rich Extracts of Red Beet (*Beta vulgaris* L.)

**DOI:** 10.3390/ijms23031245

**Published:** 2022-01-23

**Authors:** Katarzyna Sutor-Świeży, Michał Antonik, Justyna Proszek, Boris Nemzer, Zbigniew Pietrzkowski, Łukasz Popenda, Tomasz Świergosz, Sławomir Wybraniec

**Affiliations:** 1 Department C-1, Faculty of Chemical Engineering and Technology, Cracow University of Technology, ul. Warszawska 24, 31-155 Cracow, Poland; katarzyna.sutor@doktorant.pk.edu.pl (K.S.-Ś.); michal3antonik@gmail.com (M.A.); proszekjustyna@gmail.com (J.P.); tomasz.swiergosz@pk.edu.pl (T.Ś.); 2 Research and Analytical Center, VDF FutureCeuticals, Inc., 2692 N. State Rt. 1-17, Momence, IL 60954, USA; bnemzer@futureceuticals.com; 3 Food Science & Human Nutrition, University of Illinois at Urbana-Champaign, 260 Bevier Hall, 905 S. Goodwin Ave., Urbana, IL 61801, USA; 4 Discovery Research Lab, VDF FutureCeuticals, Inc., 23 Peters Canyon Rd., Irvine, CA 92606, USA; zb@futureceuticals.com; 5 NanoBioMedical Centre, Adam Mickiewicz University Poznan, ul. Wszechnicy Piastowskiej 3, 61-614 Poznan, Poland; lukasz.popenda@amu.edu.pl

**Keywords:** dehydrogenation, decarboxylation, xanbetanin, neobetanin, red beet root, colorants, betanin, betalain-rich extract, decarboxy-betacyanins, dehydrogenated betacyanins

## Abstract

Betacyanins are a group of water-soluble red-violet compounds containing nitrogen in their structure. These are biosynthesized in red beetroot (*Beta vulgaris* L.), a widely consumed vegetable that contains significant amounts of nutritious and bioactive compounds which are also found in dietary supplements. This contribution presents results of betacyanin thermal oxidation (resulting in dehydrogenation) interrelated with decarboxylation in selected acetate/phosphate buffers at pH 3–8 and at 85 °C, which may be of particular significance for formulation and performance of foods. Most of the reaction products were detected at the highest concentrations in the acidic solutions (pH 3–4). The main dehydrogenation reaction pathways were monitored by LC-DAD-MS/MS and were associated with decarboxylation of the principal extract pigments, betanin/isobetanin and neobetanin, at carbon positions C-2 and C-17. Additional reactions are accompanied by the 2,15-decarboxylation processes at different dehydrogenation levels with 15-decarboxy-betanin and 2,15-bidecarboxy-betanin, structurally elucidated by NMR analysis, as the distinct indicators of this route type. For other novel pigments detected, 2,15-bidecarboxy-xanbetanin, 2,15-bidecarboxy-xanneobetanin and 2,15,17-tridecarboxy-neobetanin, additional high resolution mass spectrometric analyses were performed and confirmed their molecular formulas.

## 1. Introduction

Beetroot (*Beta vulgaris* L.) is one of the important vegetables and contains significant amounts of nutritious and bioactive compounds. One group of them are the natural pigments—betalains from which betanin is present in extracts of *B. vulgaris* roots at a level of 300–600 mg/kg of the extract [1]. Betalains are a group of water-soluble colored compounds containing nitrogen in their structure [2] divided into 2 groups—betacyanins and betaxanthins. They are synthesized by most plants of the order Caryophyllales [3], and are also found in some species of fungi of the genera *Amanita* and *Hygrocybe* [4,5]. At present, 187 betacyanins from natural sources have been identified [6].

The chemical synthesis of betalains is difficult [7,8]. That is why their common source is a natural raw material, especially the roots of *B. vulgaris* [9,10]. It belongs to the top 10 vegetables containing antioxidants [10,11] which is the approved source of betalains as additives used in food in Code of Federal Regulations of United States (21CFR73.40) and European Union code (E162) [12].

Beetroot extract and its ingredients are used as dietary supplements [13]. In vitro studies as well as animal models in vivo [14,15] have shown promise for the use of beetroot extract’s antioxidant and anti-inflammatory properties in the case of chronic inflammation, liver diseases [16], arthritis [17], and even with diseases associated with cancer [18,19]. Many independent studies have confirmed the high antioxidant activity of betacyanins [20,21,22,23], and some data suggests a correlation between antioxidant activity and betacyanin content in beetroot juice [20]. Additionally, via preclinical studies conducted in rats, it was shown that the consumption of approximately 8 mL of beet juice per 1 kg of body weight for 28 days ameliorated xenobiotic induced liver DNA damage by reducing lipid peroxidation and protein oxidation [1].

Compounds of the betalain family may be degraded under the influence of external factors, including temperature, which is their main disadvantage in terms of their use in the food or pharmaceutical industry [24]. The stability of betalains in solutions is also limited by such environmental factors as pH, water activity, light, the presence of oxygen, the presence of enzymes, compounds with antioxidant activity, or metal cations [25,26]. Betacyanins show greater stability than betaxanthins, both at room temperature and when heated. Herbach, with his team [26], reported that increasing temperatures generates betalamic acid and neobetacyanins from betacyanins. In addition, decarboxylation and cleavage of the glycosidic group may occur [27]. In addition, improvement in stability after glycosylation is indicated [28]. The literature also points to the low stability of some acylated betalains [26,29].

Betalains show good stability in the pH range from 3 to 7, but the most optimal conditions for them are environments with a pH in the range of 4–6, and their stability increases in anaerobic conditions [29]. Betacyanins are stable during short-term heating (up to 3 min) at 80 °C [30]. Kidoń and Czapski found that during more than 3 min blanching of beetroot at 90 °C there is a 25% reduction in the content of red pigments; however, extending this time to 10 min does not significantly affect further losses [31]. The thermal degradation of betacyanins is primarily the result of their breakdown into *cyclo*-DOPA derivatives and betalamic acid derivatives, and is in most cases reversible.

The factors improving betalain stability include ascorbic acid [32], isoascorbic acid [26,33,34], chelating agents such as citric acid, and EDTA [26,35]. β-cyclodextrin and glucose oxidase, which act by adsorbing free water and removing dissolved oxygen, may also be effective [36]. Phenolic antioxidants and tocopherol did not show any stabilizing effect [34].

The first report on decarboxylated betacyanin structures in plants can be found in an article from 1970 [37] supporting the endogenous occurrence of 2-decarboxybetanidin. The preferred thermic cleavage of the carboxyl group at the C-17 position in betanidin was indicated by Minale and Piatelli [38]. After 2000, research began to indicate the possibility of obtaining new decarboxylated betacyanins which was presented, among others, in our previous publications [39,40]. The development of analytical techniques and the use of new high-performance liquid chromatographic (HPLC) columns have enabled the isolation and testing of decarboxylated derivative structures, thanks to which various thermal degradation products of betacyanins, depending on the reaction environment, have been observed. These products include mono, bi-, and tri-decarboxy-betacyanins, as well as their 2,3-(xan) or 14,15-dehydrogenated (neo) analogues. Importantly, in the aqueous environment, especially at the initial stages of degradation, other products are obtained than in the case of reactions in ethanolic solutions [33,39,40]. In addition, the physicochemical conditions of the reaction environment (pH, temperature, or the presence of metal ions) have an impact on obtained structures [41]. During the heating degradation of betacyanin-rich red beetroot extract (RBE), depending on the prevailing conditions, compounds such as 2-, 15-, and 17-decarboxy-betanin, together with their corresponding neo-derivatives as well as appropriate isomers, may be formed followed by their bi- or tri- decarboxy analogues [6,21,42]. Based on these assumptions, it can be concluded that the mechanism of betacyanin degradation may be different, and its variants were initially presented in our publications [41,43,44,45]. In the course of many years of work on decarboxylated compounds, the above-mentioned and many other structures have been confirmed by LC-MS and/or NMR methods [6,46,47].

This report presents results of thermal dehydrogenation studies on betacyanins present in a specifically purified highly concentrated betalain-rich extract (BRE) [45] and focuses on the possible directions of degradation routes of the pigments in betacyanin-rich red beetroot extract [45] during heating depending on the process conditions, such as used buffer, pH, temperature, heating time, and the addition of stabilizing agents. The first tentative structures formed by decarboxylation of the main pigment in BRE, betanin, and its diastereomer, were established by means of liquid chromatography coupled to diode array detection and electrospray ionization tandem mass spectrometry (LC-DAD-ESI-MS/MS) [45]. In the extract, two new isomeric bidecarboxylated betanins were tentatively identified. A high rate of generation of 2-decarboxy-betanin/-isobetanin, which is present in the BRE extract at a very low level, was observed, which was dependent on the starting concentration of the BRE substrate. The bidecarboxylated derivatives were generated at a higher rate mostly from 17-decarboxy-betanin/-isobetanin as well as 15-decarboxy-betanin by further decarboxylation at carbon C-2 [47].

## 2. Results and Discussion

Previous studies on betanin degradation in heated red beet extracts resulted in identification of the principal decarboxy-betanins based on 2- and 17-decarboxylation [26,33,39]. Recent studies have broadened the palette of the compounds and tentatively reported new bidecarboxylated betanins in heated red beetroot extracts which enabled construction of first possible decarboxylation routes [47]. However, no deeper research was performed on oxidation (dehydrogenation) pathways during heating of red beet extracts which could be combined with the decarboxylation steps, and only several dehydrogenated products were tentatively detected based on 2,17-decarboxylation routes [26,39]. In this report, identification of 15-decarboxy-betanin **4** and 2,15-bidecarboxy-betanin **11** by NMR and high-resolution mass spectrometry enabled further construction of alternative dehydrogenation pathways. Taking into account that especially 2,15-bidecarboxy-betanin can be present at higher quantities in processed *B. vulgaris* juices and extracts [47], the other pathways became possible to be followed.

**Figure 1:**
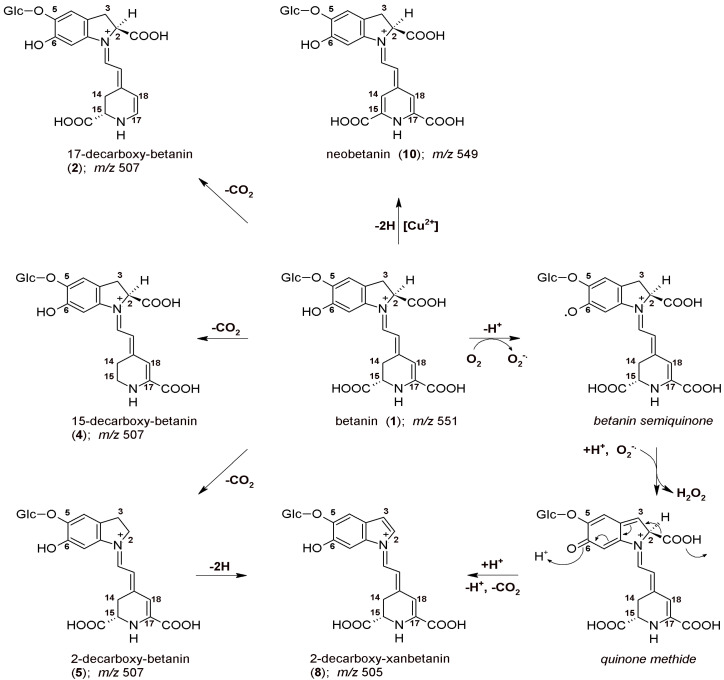
The initial mechanism of betanin autoxidation based on the formation of quinone methide which transforms into the xan-derivative with additional 2-decarboxylation [42,44]. Another betanin oxidation possibility by catalysis with Cu^2+^ resulting in generation of neobetanin [46] as well as possible positions of decarboxylation are presented.

In this context, it is necessary to mention that due to previous oxidation structural studies on betacyanins, with the use of enzymatic [40] and chemical agents [44], several dehydrogenation pathways have been better recognized. Furthermore, the key oxidation products were isolated and identified by NMR confirming the 2,17-decarboxylation routes [46]. The initial mechanism of betanin oxidation as well as principal directions of decarboxylation are presented in Figure 1. The main betanin oxidation mechanism is based on the formation of the quinone methide which transforms into the xan-derivative with additional 2-decarboxylation. This reaction can be initiated by autoxidation during the heating. In addition, after initial decarboxylation of betanin at carbon C-2, the reaction follows a similar oxidation pathway [42,44]. Another betanin oxidation mechanism is also possible by catalysis with heavy metal cations, especially Cu^2+^, resulting in generation of neobetanin (Figure 1) [46]. As demonstrated previously [46], 2-decarboxylation is accompanied mainly by 17-decarboxylation, however, the impact of initial 15-decarboxylation of betanin should be also taken into account, as should the formation of high quantities of 2,15-bidecarboxy-betanin during the heating. This pathway seems to be equally important for considering the whole betanin reaction scheme. Therefore, in this report, the dehydrogenation steps in heated concentrated betalain-rich extract involving transformations of the 15-decarboxylated derivatives are presented for the first time.

### 2.1. Chromatographic and Mass Spectrometric Monitoring of the Products Generated during the BRE Heating Experiments

**Figure 2:**
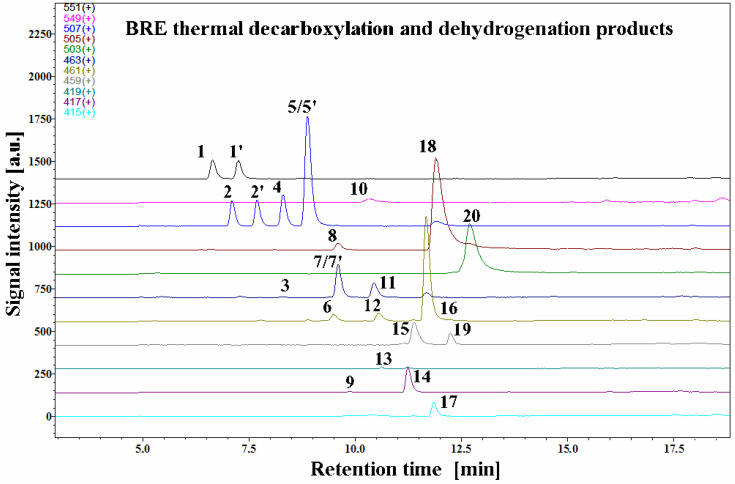
Chromatographic LC-MS profiles of selected ions of betanin as well as its decarboxylated and dehydrogenated derivatives generated in betalain-rich extract after the 45-min heating experiments in acetate/phosphate buffers at 85 °C (Compound numbers as in Table 1).

The LC-MS selected ion chromatograms present in Figure 2 depict typical betanin as well as its decarboxylated and dehydrogenated derivative profiles in a betalain-rich extract/concentrate (BRE) after the 45-min heating experiment in acetate buffers at pH 3 and 85 °C.

Interpretation of the LC-DAD and LC-MS spectra enabled identification of known as well as novel betanin derivatives observed during all the experiments (Table 1). Recently, an important simplification of the naming of betacyanin derivatives was proposed [6] to substitute the phrase “2,3-dehydro” by “xan” in the trivial name of the 2,3-dehydrogenated betacyanins in reference to the “neo” prefix used to substitute the phrase “14,15-dehydro”.

All the detected degradation products of the pigments were less polar than their corresponding precursors. The dominant presence of unreacted betanin **1** and its isoform **1′** with substantial presence of neobetanin **10** resembles the starting betalainic profile from a previous research [45]. Additional similarities are in distinct signals of very well separated 17-decarboxy-betanin/-isobetanin **2/2′** and 15-decarboxy-betanin **4** (hitherto, detected only tentatively) as well as 2-decarboxy-xanbetanin **8** and 2-decarboxy-xanneobetanin **20**, the latter pigment being the most hydrophobic product of betanin transformation. In Section 2.5, the final structural results for 15-decarboxy-betanin **4** obtained by NMR are presented.

In addition, four highly abundant derivatives, especially at pH 3, were detected in this study: 2-decarboxy-betanin/-isobetanin **5/5′**, 2,17-bidecarboxy-betanin/-isobetanin **7/7′**, 2-decarboxy-neobetanin **18**, and 2,17-bidecarboxy-neobetanin **16**. For the identification of known derivatives, a series of already known decarboxylated and dehydrogenated betanin standards was used in the study [33,39,41,42,44,45,46].

**Figure 3:**
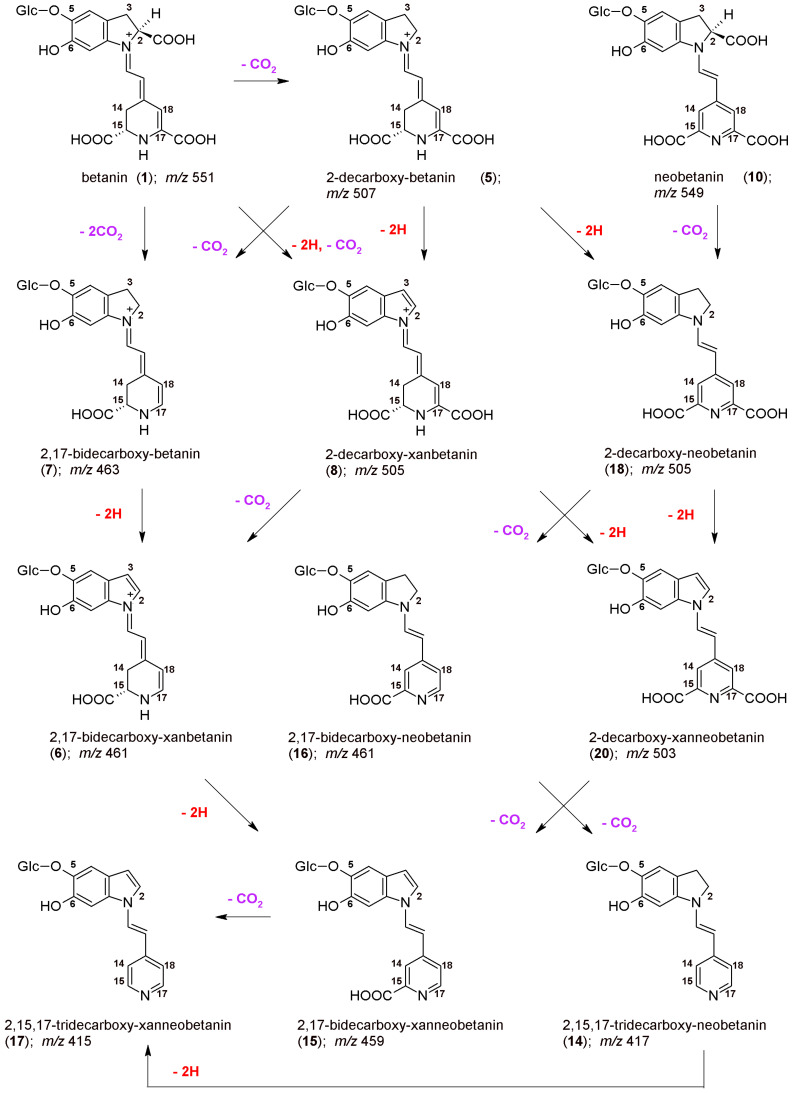
The proposed principal oxidation (**-2H**) pathways occurring during the BRE heating starting from betanin and neobetanin through the main 2,17-decarboxylation routes.

The chemical formulas as well as the proposed reaction pathways starting from betanin and neobetanin through the main 2,17-decarboxylation routes are depicted in Figure 3. They are based on the identification of **2/2′**, **5/5′**, **7**, **8**, **16**, **18**, and **20** in the reaction mixtures, but also on detection of 2,17-bidecarboxy-xanbetanin **6** as well as doubly oxidized 2,17-bidecarboxy-xanneobetanin **15** and completely decarboxylated derivatives, 2,15,17-tridecarboxy-neobetanin **14** and 2,15,17-tridecarboxy-xanneobetanin **17**. Only a minute signal for non-oxidized 2,15,17-tridecarboxy-betanin **13** was noticed (Table 1), possibly because of co-occurrance of the oxidation processes [42,44,46].

Interestingly, the presence of 2-decarboxybetanidin was not detected pointing to the stability of the glucosidic linkage under the acidic conditions.

Further inspection of chromatograms revealed also 2,15-bidecarboxy-betanin **11** (previously tentatively identified [47]), a key reaction product in further discussion on alternative betanin oxidation pathways in the following sections. In this contribution, its identity was confirmed by NMR analysis for the first time (Section 2.5). The lack of the carboxyl moiety at carbon C-15 implicates the lack of the chirality at this position, therefore, only single forms of the pigments **4** and **11** as well as all the neo-derivatives were detected in the chromatograms, which supports the pigment identification.

Other 2,15-bidecarboxylated derivatives: 2,15-bidecarboxy-xanbetanin **12** as well as doubly oxidized 2,17-bidecarboxy-xanneobetanin **15** and 2,15-bidecarboxy-xanneobetanin **19** were detected in the chromatograms. A very small signal detected for **9** was assigned to 2,15,17-tridecarboxy-xanbetanin—a more polar isomer of **14**, based on assumption that the xan-derivatives of betanin are eluted faster than the isomeric neo-derivatives [40,42,44,46]; however, co-elution with other compounds and low intensity prevented its further determination (Table 1).

### 2.2. Influence of pH on Generation of Decarboxylated Betanins during BRE Heating

**Figure 4:**
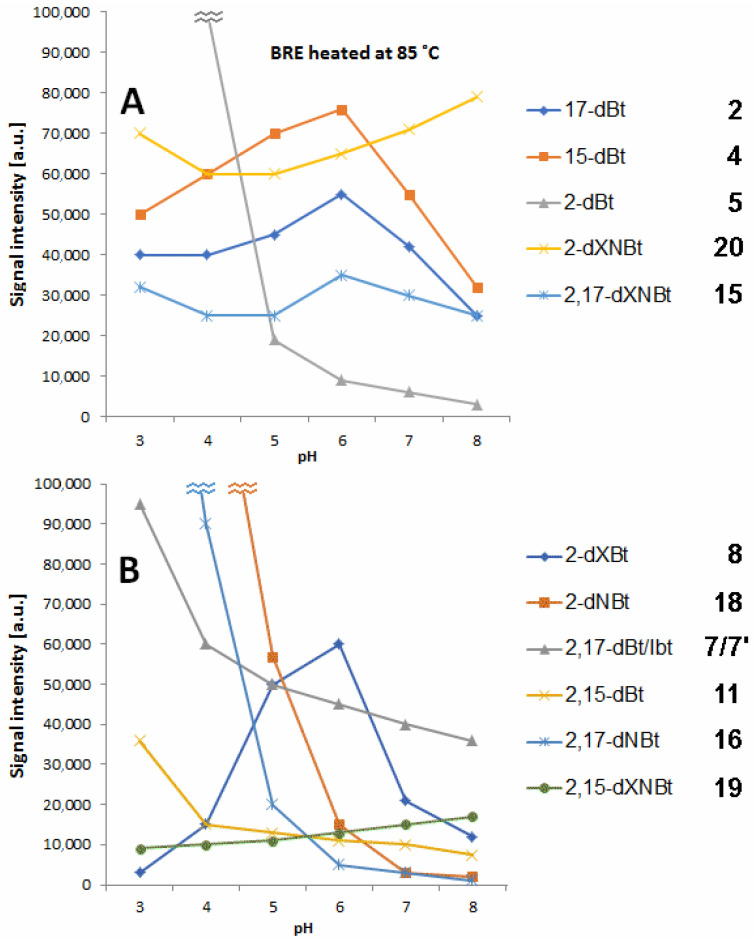
LC-MS signal levels of prominent mono- (**A**) and bidecarboxylated (**B**) betanin derivatives (**2**; **4**; **5** and **7/7′**; **11**, respectively) and dehydrogenated betanins (**8**, **15**, **16,** and **18**) as well as most hydrophobic xanneobetanins (**19** and **20**) detected after 45 min extract heating at 85 °C in acetate/phosphate buffer solutions in dependence on pH.

In Figure 4, the profiles of prominent decarboxylated and dehydrogenated betanin derivatives detected by LC-MS in selected buffer solutions after 45 min extract heating at 85 °C in dependence on pH are presented. The levels of 15-dBt **4** and 17-dBt **2** tend to decrease at high pH (7–8) with a distinct peak at pH 6 (Figure 4). In general, the observed profiles of 15-dBt and 17-dBt in the whole tested pH range confirm their steady generation from betanin as well as their further transformation. In contrast, 2-decarboxylation effect takes place at high extent in the more acidic environment (pH 3–4).

In a previous report [47], the most decisive factor in the preferential generation of 2-dBt/-IBt **5/5′** was the concentration of the substrate (Bt/IBt **1/1′**). Another important factor was a concentration of acetic acid whose lower concentration (1 g/L) promoted the generation of **5/5′**. Earlier studies also confirmed preferential generation of **5/5′** in aqueous acidic solutions of red beet extract in contrast to ethanolic solutions which enhanced the generation of 17-dBt/-IBt **2/2′**.

Double decarboxylation results mostly in generation of 2,17-bidecarboxy-betanin/-isobetanin **7/7′** and some lower quantities of 2,15-bidecarboxy-betanin **11** (Figure 4). Similarly to 2-dBt/-IBt **5/5′**, the elevated rate of their formation is noticed at pH 3–4.

Very small quantities of 15,17-bidecarboxy-betanin **3** (Table 1 and Table 2) were detected and solely in more acidic solutions. This result confirmed data from the previous study [47] where the concentration of 15,17-dBt **3** decreased after the heating, therefore, this pigment was not meaningfully generated. It is possible that its presence resulted only from a chemical process taking place during production of the BRE extract, but it cannot be formed by heating.

High concentration of BRE enhanced the formation of 2,17-dBt **7/7′** over 2,15-dBt **11** [47]. During the heating experiments, contents of compounds **7/7′** and **11** successively differed at different acetic acid as well as BRE concentrations. At a low concentration of BRE, pigment **11** signal dominated, and this effect was more pronounced at the higher concentrations of acetic acid (2.5 g/L); however, those differences were, presumably, attributed to the matrix effect [47].

In the current study, the presence of 2,15,17-tridecarboxy-betanin **13** before heating but also after the heating experiments was not acknowledged in accordance with the previous report [45]. Nevertheless, this is in contrast to the previous complementary experiments at other conditions [47] in which it was strongly dependent on different acetic acid and BRE concentrations. Increased concentration of acetic acid enhanced the generation of pigment **13**, especially at the higher BRE concentration. However, during the heating experiment, the content of **13** increased successively at all conditions.

### 2.3. Influence of pH on Generation of Dehydrogenated Betanins during BRE Heating

Several dehydrogenated betanins are known derivatives [6] which were also detected previously in the BRE extract [45]. The most hydrophobic is 2-decarboxy-xanneobetanin **20** which, together with bidecarboxylated derivatives, presumably 2,15-bidecarboxy-xanneobetanin **19** (Figure 4) as well as 2,17-decarboxy-xanneobetanin **15** (Figure 4) are generated at higher quantities at pH 7–8.

Pigment **15** is the decarboxylated derivative of **20**, therefore, this 2-decarboxylation and dehydrogenation path is clearly deduced (Figure 3). This path starts with the generation of 2-decarboxy-xanbetanin **8** from betanin **1** and 2-decarboxy-neobetanin **18** from neobetanin **10** (Figure 3). Generation of both the derivatives is, in general, observed in acidic solutions (Figure 4); however, there are some distinct differences for the compounds. The highest rate for **18** is observed at pH 3–4 but for **8** the optimal pH range is shifted to 5–6 (Figure 4).

Another prominent derivative is 2,17-bidecarboxy-neobetanin **16** is especially visible in the heating products at pH 3–4 and its presence confirms the 2,17-decarboxylation and dehydrogenation path from betanin **1** but also 2,17-decarboxylation path from neobetanin **8** (Figure 3).

### 2.4. High Resolution Mass Spectrometric Determination of Novel Pigment Molecular Formulas

For further confirmation of the 15-decarboxylation pathway during the thermal oxidation of betanin, several 15-decarboxylated derivatives were submitted to the high-resolution mass spectrometric determination of their molecular formulas. The LC-IT-TOF analyses of **4** in the positive mode yielded high-resolution *m*/*z* 507.1603 (C_23_H_27_N_2_O_11_, calculated mass: 507.1609) supporting identification of a decarboxylated betanin, being 15-decarboxy-betanin according to a further NMR analysis. Subsequent collision-induced fragmentation experiments (obtained by the triple quadrupole and the high-resolution IT-TOF mass spectrometers) of the protonated ions [M + H]^+^ of **4** revealed MS^n^ fragmentation pathways (Table 2) associated with the neutral loss of the glucosyl moiety (507 − 162 = 345) as well as formic acid (345 − 46 = 299) with additional detachment of carbon dioxide (299 − 44 = 255) or formic acid (299 − 46 = 253). Further fragmentation of these decarboxylated chromophoric systems was indicated by a loss of acetonitrile and detection of ions at *m*/*z* 214 and 212 Da (255 − 41 = 214 and 253 − 41 = 212, respectively) or a neutral loss of C_3_H_5_N (255 − 55 = 200). Further ions detected at *m*/*z* 176, 162, 150, and 132, presumably resulted from a neutral loss of pyridine (255 − 79 = 176), methylated pyridine (255 − 93 = 162) and 4-vinylpyridine (255 − 105 = 150) with subsequent dehydration (150 − 18 = 132) (Table 2). In the positive mode, the high-resolution *m*/*z* values were confirmed for the fragmentation ion of **4**, 345.1091 (C_17_H_17_N_2_O_6_, calculated mass: 345.1081).

For other novel pigments detected, 15,17-bidecarboxy-betanin **3**. 2,15-bidecarboxy-betanin **11**, 2,15-bidecarboxy-xanbetanin **12**, 2,15,17-tridecarboxy-neobetanin **14**, 2,15-bidecarboxy-xanneobetanin **19**, additional high resolution mass spectrometric analyses on an LCMS-IT-TOF system confirming the molecular formula were performed in the positive ion mode (Table 2).

For pigments **3** and **11**, the HRMS analyses yielding *m*/*z* 463.1722 and 463.1720, respectively (C_22_H_27_N_2_O_9_, calculated *m*/*z*: 463.1711), supported the presence of molecular formulas of bidecarboxylated betanins. The observed fragmentation pathway for **3** afforded signals at *m*/*z* 301 (Table 2), indicating detachment of the glucosyl moiety (463 − 301 = 162 Da) as well as at *m*/*z* 257 (-CO_2_) and 255 (-HCOOH).

Subsequent collision-induced fragmentation experiments of the protonated ions [M + H]^+^ of **11** revealed MS^n^ fragmentation pathways (Table 2) associated with the neutral loss of the glucosyl moiety (463 − 162 = 301) as well as carbon dioxide (301 − 44 = 257) or formic acid (301 − 46 = 255). Further fragmentation of these decarboxylated chromophoric systems was indicated mainly by a neutral loss of C_3_H_5_N (257 − 55 = 202) as well as further ions detected at *m*/*z* 164, 162, 150 and 132, presumably resulting from a neutral loss of methylated pyridine (257 − 93 = 164 and 255 − 93 = 162, respectively) and 4-vinylpyridine (255 − 105 = 150) with subsequent dehydration (150 − 18 = 132) (Table 2). In the positive mode, the high-resolution *m*/*z* values were confirmed for the fragmentation ion of **11**, 301.1192 (C_16_H_17_N_2_O_4_, calculated mass: 301.1183).

For pigment **12**, the HRMS analyses yielding *m*/*z* 461.1547 (C_22_H_25_N_2_O_9,_ calculated *m*/*z*: 461.1555) supported a molecular formula of bidecarboxylated xanbetanin or neobetanin. The observed fragmentation pathway afforded signals at *m*/*z* 299 (Table 2), indicating detachment of the glucosyl moiety (461 − 299 = 162 Da) as well as at *m*/*z* 255 (-CO_2_) and 253 (-HCOOH).

Because of the presence of 2,15-bidecarboxy-betanin **11** in the heating products, the 2,15-bidecarboxylation in **12** is suggested. Similar retention of **11** and **12** suggests a presence of xanbetanin derivative in contrast to more hydrophobic neobetanin derivatives [40,42,44,45,46].

For pigment **14**, the HRMS analyses yielded *m*/*z* 417.1669 (C_21_H_25_N_2_O_7,_ calculated *m/z*: 417.1656) indicating the presence of 2,15,17-tridecarboxy-neobetanin as the more hydrophobic isomer from the pair of xan- and neo-derivatives (**9** and **14**, respectively). This pigment has never been detected in products of betanin oxidation [40,42,44,45,46] but, rather, after heating degradation [39,40]. The fragmentation of the [M + H]^+^ ion resulted in detection of signals at *m/z* 255 (glucosyl detachment) and 237 (-H_2_O) (Table 2).

Determination of *m*/*z* value for **19** observed at 459.1391 (C_22_H_23_N_2_O_9,_ calculated *m*/*z*: 459.1398) indicated a presence of a bidecarboxylated xanneobetanin which is a doubly dehydrogenated derivative. This compound is an isomer of the already well-known 2,17-bidecarboxy-xanneobetanin **15** generated during oxidation experiments [42,44,45,46] and its longer retention time suggests a presence of a more hydrophobic 2,15-decarboxylation pattern directly indicating a presence of 2,15-bidecarboxy-xanneobetanin **19**. The fragmentation of the [M + H]^+^ ion resulted in detection of signals at *m*/*z* 297 (glucosyl detachment) as well as at *m*/*z* 253 (-CO_2_) and 251 (-HCOOH) (Table 2).

### 2.5. NMR Structural Elucidation of 15-Decarboxy-Betanin ***4*** and 2,15-Bidecarboxy-Betanin ***11***

**Figure 5:**
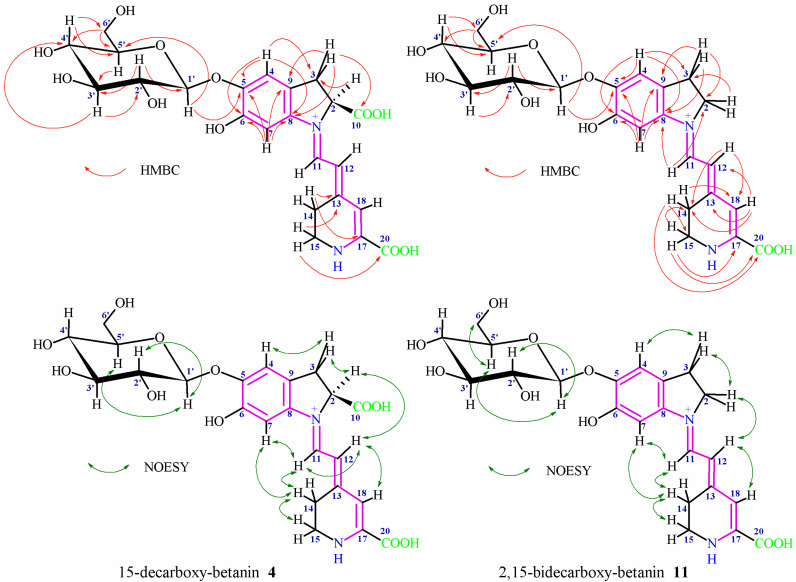
Important HMBC and NOESY NMR correlations supporting the structure elucidation of 15-decarboxy-betanin **4** and 2,15-bidecarboxy-betanin **11** present in the BRE extract as well as generated during its heating.

The characteristic NMR signals (Figures S1–S4) of the aglycone and glucose moiety in **4** and **11** confirmed the presence of a mono- and bi-decarboxylated betanin (Figure 5), respectively, (Table 3) [33,46]. Good solubility of **4** in D_2_O enabled its analysis in less destructive environment [43] whereas lower solubility of **11** in D_2_O required application of CD_3_OD acidified with d-TFA. This enabled complete solubilization of **11** as well as obtaining stable zwitterionic systems with narrowed signals [48] with no degradation of the pigment detected. The individual coupled ^1^H-spin systems of the aglycone (H-2 or H-2ab, H-3ab; H-11, H-12; H-14ab, and H-15ab) were assigned in ^1^H NMR, COSY, and TOCSY spectra. The three-spin system observed for H-2/H-3ab in **4** indicated the presence of the carboxyl moiety at C-2 similar to betanin, this way excluding the decarboxylation at carbon C-2. In contrast, the three-spin system observed for H-2ab/H-3ab in **11** confirmed the decarboxylation at carbon C-2.

In both the pigments **4** and **11**, the doublets for the H-11 and H-12 protons (very broad for **4**) were readily distinguishable by their low- and high-field shifts, respectively. In contrast to **11**, a very broad signal for H-18 in **4** was observed by ^1^H NMR and was detected for freshly prepared solution in D_2_O of the pigment, thus avoiding the fast deuterium exchange [48]. The four-spin system (H-15ab/H-14ab) showed easily distinguishable cross-peaks in the COSY and TOCSY spectra; however, in contrast to betanin, the presence of two protons at carbon C-15 indicated the decarboxylation position at carbon C-15. The dihydroindolic system was assigned by HSQC correlations of H-2 or H-2ab, H-3ab, H-4 and H-7 with their respective carbons. In the dihydropyridinic system, correlations of H-14ab, H-15ab, and H-18 with their respective carbons in the HSQC spectra were visible.

In **4**, the correlations of C-3 to H-2/H-4, C-5 to H-4/H-7, C-6 to H-4/H-7, C-8 to H-3ab/H-4/H-7, C-9 to H-3ab/H-7, and C-10 to H-3ab (the dihydroindolic system) as well as the correlations of C-13 to H-14/H-15, C-17 to H-14, C-18 to H-12/H-14 and C-20 to H-15 (the dihydropyridinic system) were determined by HMBC (Figure 5, Table 3).

In **11**, the correlations of C-2 to H-3/H-11, C-3 to H-2/H-4, C-5 to H-4/H-7, C-6 to H-4/H-7, C-8 to H-3ab/H-4/H-7/H-11, and C-9 to H-3ab/H-7 (the dihydroindolic system) as well as the correlations of C-13 to H-18, C-14 to H-12/H-15/H-18, C-15 to H-14, C-17 to H-15, C-18 to H-12/H-14, and C-20 to H-14/H-15 (the dihydropyridinic system) were determined by HMBC (Figure 5, Table 3).

In **4** and **11**, the ^1^H and ^13^C chemical shifts for the protons and their corresponding carbons in the glucose moieties were assigned by the COSY, TOCSY, HSQC, and HMBC correlations which clearly ascertained the sugar ring systems (Figure 5, Table 3). The presence of the anomeric proton H-1′ indicating a sugar unit by its characteristic downfield shifts was readily observed. The position of the glycosidic bond at the phenolic carbon C-5 was confirmed by the HMBC correlation of the anomeric proton H-1′ with carbon C-5 as well as it was indicated by the downfield shift for the proton H-4 in relation to H-7 [33,48]. The coupling constant via the three vicinal bonds ^3^*J*_1′-2′_ (7.3–7.4 Hz in **4** and **11**) indicates the presence of a *β*-glycosidic link between the aglycone and the glucoside moiety of this pigment. A definitive evidence of the lack of acylation at C-6′ carbon was provided by the position of the H-6′ab protons signal.

Additional data observed in the NOESY spectra confirmed the key correlations (Figure 5) between H-7, H-11 and H-14a/b which together with correlations of H-12 with H-2 and H-18 indicated the (*E*)-configuration for C(12)=C(13) and s-*trans* conformation for the dienyl moiety N(1)=C(11)-C(12)=C(13) in the most abundant stereoisomers in **4** and **11 [48]**. Additional correlations were observed between the newly originated methylene protons H-15a/b (in comparison to betanin) with H-14a/b as well as between H-2, H-3 and H-4 and between selected H atoms of the glucosyl moiety. Above analysis completed the structure identification of 15-decarboxy-betanin **4** and 2,15-bidecarboxy-betanin **11**. 

### 2.6. Alternative 2,15-Decarboxylation Pathway in Thermal Oxidation of Betanin

The 2,17-decarboxylation path of betanin and neobetanin degradation during heating is the most probable direction because of additional possibility of simultaneous decarboxylation and oxidation of the molecule at carbon C-2,3 (dehydrogenation) and subsequent transformations of the intermediate products followed by decarboxylation step at C-17.

**Figure 6:**
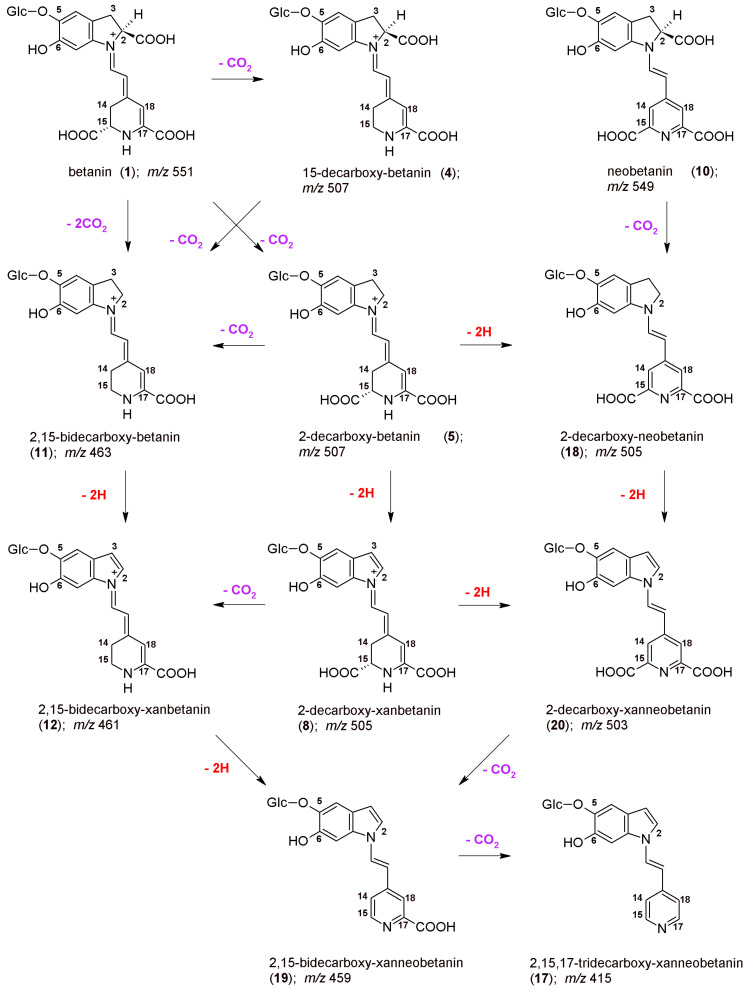
The proposed oxidation (**-2H**) pathways occurring during the BRE heating starting from betanin and neobetanin through the main 2,15-decarboxylation routes, based on chromatographic, mass spectrometric, and NMR studies of the reaction products.

However, we can also assign another pathway (Figure 6) starting mainly from the 15-decarboxylation of betanin **1**, resulting in generation of the key 15-decarboxy-betanin **4** derivative as well as 2,15-bidecarboxy-betanin **11** (with confirmed structures by NMR in this study). Subsequent formation of 2,15-bidecarboxy-xanbetanin **12** and especially distinctive quantities of 2,15-bidecarboxy-xanneobetanin **19** (Figure 4) supports this pathway which is completed with 2-decarboxylation of neobetanin **10** as well as further dehydrogenation at carbon C-2,3 (resulting in formation of 2-dXNBt **20**) and 15-decarboxylation leading again to **19** (Figure 6). Final generation of the end chromophoric structure of 2,15,17-tridecarboxy-xanneobetanin **17** seems to be attained by one-step 17-decarboxylation of **19** but also 15,17-decarboxylation of **18** (Figure 3) leading to 2,15,17-tridecarboxy-neobetanin **14** followed by dehydrogenation at carbon C-14,15.

## 3. Materials and Methods

### 3.1. Reagents

Formic acid, acetic acid, LC-MS grade methanol and water, and HPLC grade acetone and buffer solutions were obtained from Sigma Chemical Co. (St. Louis, MO, USA).

### 3.2. Heating Experiments

Betalain-rich extract (BRE) was obtained from FutureCeuticals, Inc. (Momence, IL, USA) [45]. BRE aqueous stock solution (50 mL) was prepared at a concentration of 0.75 g/L and was 10x diluted in microplate wells up to 200 μL. Each well contained 20 μL of acetate/phosphate buffers at pH 3–8 (20 mM). These samples were heated at 85 °C in a thermostat for 1 h and were monitored by spectrophotometry in a microplate reader Tecan Infinite 200 (Tecan Austria GmbH, Grödig/Salzburg, Austria). During the experiments, additional aliquots (20 μL) of the heated samples were taken for LC-DAD-ESI-MS/MS analyses after 20x dilution. All the experiments were performed in triplicate.

### 3.3. Preparation of 15-Decarboxy-Betanin ***4*** and 2,15-Bidecarboxy-Betanin ***11*** from BRE Extract

For the NMR study, 15-decarboxy-betanin **4** and 2,15-bidecarboxy-betanin **11** were derived directly from the BRE extract by chromatography. Eight grams of the extract was dissolved in 12 L of water and was initially purified by flash chromatography on a column 40 mm × 50 mm filled with Sepra™ ZT-SAX 30 μm Polymer, 85-Å (Phenomenex, Torrance, CA, USA). Further separation and isolation of the pigment was performed on a HPLC semipreparative column Synergi Hydro-RP 250 mm × 30 mm i.d., 10 μm (Phenomenex) with a 20 mm × 25 mm i.d. guard column of the same material (Phenomenex). A gradient system consisting of 1% aqueous formic acid (solvent A) and acetone (solvent B) was used as follows: 0 min, 12% B; increasing to 10 min, 14% B; increasing to 20 min, 16% B; increasing to 30 min, 18% B; increasing to 40 min; 80% B. The injection volume was 20 mL with a flow rate of 30 mL/min. Detection was performed using a PDA UV/Vis detector at 538, 505, 480, and 440 nm; at column temperature of 22 °C. The eluates were pooled and concentrated under reduced pressure at 25 °C and finally freeze-dried. All the solutions were concentrated in rotary evaporators at 25 °C under reduced pressure to remove the organic solvent and stored at −20 °C for further studies.

### 3.4. LC-DAD-ESI-MS/MS Analyses

For qualitative as well as quantitative analyses of the samples, a low-resolution LC-MS-8030 mass spectrometric system (Shimadzu, Kyoto, Japan) coupled to LC-20ADXR HPLC pumps, an injector model SIL-20ACXR, and a PDA detector (photo diode array) model SPD-M20A, all controlled with LabSolutions software, version 5.60 SP1 (Shimadzu) was applied. The samples were eluted through a chromatographic column (150 mm × 4.6 mm i.d., 5.0 μm, Kinetex C18) preceded by a guard column of the same material (Phenomenex, Torrance, CA, USA). The injection volume was 50 μL, and the flow rate was 0.5 mL/min. The column was thermostated at 40 °C.

Sample solutions were pumped through the column under the following elution gradient system (System 1) composed of 2% aqueous formic acid (A) and pure methanol (B) as follows: 0 min, 10% B; increasing linearly to 12 min, 40% B; increasing linearly to 15 min, 60% B; increasing linearly to 19 min, 90% B. Columns were thermostated at 40 °C. The injection volume was 10 μL, and the flow rate was 0.5 mL/min. The detection was performed in the full PDA range and at selected wavelengths (440, 480, 505, and 540 nm). The ionization electrospray source operated in positive mode (ESI+) at an electrospray voltage of 4.5 kV, capillary temperature at 250 °C and using N_2_ as a sheath gas. The LC-MS system was controlled with LabSolutions software, version 5.60 SP1 (Shimadzu), recording total ion chromatograms, mass spectra, ion chromatograms in selected ion monitoring mode (SIM), and the fragmentation spectra. Argon was used as the collision gas for the collision-induced dissociation (CID) experiments. The relative collision energies for MS/MS analyses were set at −35 V.

### 3.5. Chromatographic Analyses with Detection by Ion-Trap Time-Of-Flight System (LCMS-IT-TOF)

The mass spectrometer (Shimadzu) with electrospray ionization method (ESI) was applied to record all mass spectra. It was coupled to the HPLC Prominence (Shimadzu). Compounds were separated on a 50 mm × 2.1 mm i.d., 1.9 μm Shim Pack GISS C18 column (Shimadzu) thermostated at 40 °C. Samples were dosed in a volume of 2 μL and the flow rate was 0.2 mL/min. The separation of the analytes was performed in the same gradient systems as in the case of LC-DAD-ESI-MS/MS. Parameters of LCMS-IT-TOF spectrometer were set as follows: curved desolvation line (CDL) and heat block temperature 230 °C, nebulizing gas flow rate 1.5 L/min and capillary voltage 4.5 kV. Positive ion mode with mass range within 100–2000 Da was applied for recording all mass spectra. Collision energy was in the range of 12–50% depending on the structure of compounds. The Formula Predictor within the LCMS Solution software was used for elaboration of results obtained in high resolution mass spectrometry experiments (HRMS). Only empirical formula with a mass error below 5 ppm were taken into account.

### 3.6. NMR Experiments

The NMR data of **4** were recorded on a Bruker Avance III 600 spectrometer (Bruker Corp., Billerica, MA, USA) equipped with a 5 mm TBI probe head in non-acidified D_2_O at temperature of 298 K. The NMR spectra of **11** were acquired on a Bruker Avance III 700 spectrometer (Bruker Corp., Billerica, MA, USA) using a QCI CryoProbe at 295 K in CD_3_OD acidified by d-trifluoroacetic acid.

All 1D (1H) and 2D NMR (COSY, HSQC, HMBC, TOCSY, and NOESY (gradient enhanced)) measurements were performed using standard pulse sequences and acquisition parameters. The residual water peak for experiments carried out in D_2_O was suppressed using the low-power presaturation. Chemical shifts were referred to internal 3-(trimethylsilyl)-2,2,3,3-tetradeuteropropionic acid (TMSP-d_4_) (δ_H_ = 0.00 ppm, δ_C_ = 0.0 ppm) or residual CD_3_OD (δ_H_ = 3.31 ppm, δ_C_ = 49.0 ppm).

## 4. Conclusions

This is the first report on the generation of dehydrogenated betanins in a *B. vulgaris* betalain-rich extract heated in typical buffered solutions with addition of citrates and EDTA. The main dehydrogenation reaction pathways are associated with decarboxylation of the principal extract constituents, betanin/isobetanin and neobetanin, at carbon positions C-2 and C-17. Additional reactions are accompanied by the 2,15-decarboxylation processes at different dehydrogenation levels with 2,15-decarboxy-betanin as the distinct indicator of this route type. Generated betanin derivatives might have a strong influence on the bioactivities of *B. vulgaris* products and can be used for various food applications with new health-promoting potentials and colorant properties.

## Figures and Tables

**Table 1:** Chromatographic, spectrophotometric, and mass spectrometric data of detected betanin-based decarboxylated and dehydrogenated derivatives in the betalain-rich extract (BRE) heated in acetate/phosphate buffers at 85 °C.

No.	Pigment	Abbreviation	t_R_	λ_max_	*m/z*
			[min]	[nm]	[M + H]^+^
**1**	betanin	Bt	6.6	536	551
**2**	17-decarboxy-betanin	17-dBt	7.1	505	507
**1′**	isobetanin	IBt	7.3	536	551
**2′**	17-decarboxy-isobetanin	17-IdBt	7.7	505	507
**3**	15,17-bidecarboxy-betanin ^a^	15,17-dBt	8.3	494	463
**4**	15-decarboxy-betanin	15-dBt	8.3	527	507
**5/5′**	2-decarboxy-betanin/-isobetanin	2-dBt	8.9	533	507
**6**	2,17-bidecarboxy-xanbetanin ^a^	2,17-dXBt	9.5	460	461
**7/7′**	2,17-bidecarboxy-betanin/-isobetanin	2,17-dBt/-IBt	9.6	507	463
**8**	2-decarboxy-xanbetanin ^a^	2-dXBt	9.6	446	505
**9**	2,15,17-tridecarboxy-xanbetanin ^a^	2,15,17-dXBt	9.9	-	417
**10**	neobetanin	NBt	10.3	468	549
**11**	2,15-bidecarboxy-betanin	2,15-dBt	10.4	532	463
**12**	2,15-bidecarboxy-xanbetanin ^a^	2,15-dXBt	10.6	478	461
**13**	2,15,17-tridecarboxy-betanin ^a^	2,15,17-dBt	10.7	503	419
**14**	2,15,17-tridecarboxy-neobetanin ^a^	2,15,17-dNBt	11.3	442	417
**15**	2,17-bidecarboxy-xanneobetanin	2,17-dXNBt	11.4	407	459
**16**	2,17-bidecarboxy-neobetanin ^a^	2,17-dNBt	11.7	459	461
**17**	2,15,17-tridecarboxy-xanneobetanin	2,15,17-dXNBt	11.9	394	415
**18**	2-decarboxy-neobetanin	2-dNBt	12.0	480	505
**19**	2,15-bidecarboxy-xanneobetanin ^a^	2,15-dXNBt	12.3	427	459
**20**	2-decarboxy-xanneobetanin	2-dXNBt	12.7	422	503

^a^—Tentatively identified.

**Table 2:** High-resolution mass spectrometric data obtained by IT-TOF technique for novel decarboxylated and dehydrogenated betacyanins formed during BRE heating experiments in acetate/phosphate buffer at 85 °C.

No.	Pigment and Fragmentation Ion ^a^	[M + H]^+^ Molecular Formula	[M + H]^+^ Observed	[M+ H]^+^ Predicted	Error [mDa]	Error [ppm]	MS^2^ Ions
**3**	15,17-bidecarboxy-betanin	C_22_H_27_N_2_O_9_	463.1722	463.1711	1.1	2.37	301
	nl: -Glc	C_16_H_17_N_2_O_4_	301.1194	301.1183	1.1	3.65	257; 255
**4**	15-decarboxy-betanin	C_23_H_27_N_2_O_11_	507.1603	507.1609	−0.6	−1.18	345
	nl: -Glc	C_17_H_17_N_2_O_6_	345.1091	345.1081	1.0	2.90	299; 255; 253; 214; 212; 200; 176; 162; 150; 132
**11**	2,15-bidecarboxy-betanin	C_22_H_27_N_2_O_9_	463.1720	463.1711	0.9	1.94	301
	nl: -Glc	C_16_H_17_N_2_O_4_	301.1192	301.1183	0.9	2.99	257; 255; 202; 164;162; 150; 132
**12**	2,15-bidecarboxy-xanbetanin	C_22_H_25_N_2_O_9_	461.1547	461.1555	−0.8	−1.73	299
	nl: -Glc	C_16_H_15_N_2_O_4_	299.1019	299.1026	−0.7	−2.34	255; 253
**14**	2,15,17-tridecarboxy-neobetanin	C_21_H_25_N_2_O_7_	417.1669	417.1656	1.3	3.12	255
	nl: -Glc	C_15_H_15_N_2_O_2_	255.1138	255.1128	1.0	3.92	237
**19**	2,15-bidecarboxy-xanneobetanin	C_22_H_23_N_2_O_9_	459.1391	459.1398	−0.7	−1.52	297
	nl: -Glc	C_16_H_13_N_2_O_4_	297.0861	297.0869	−0.8	−2.69	253; 251

^a^ nl—neutral losses from [M + H]^+^.

**Table 3:** The NMR Data (Figures S1–S4) of analyzed 15-decarboxy-betanin **4** and 2,15-bidecarboxy-betanin **11** isolated from the BRE extract as well as generated during its heating. Important HMBC and NOESY correlations for **3** and **8** are depicted in Figure 5.

	15-Decarboxy-Betanin 4 (D_2_O)			2,15-Bidecarboxy-Betanin 11 (CD_3_OD/d-TFA)	
No.	^1^H NMR ^a^	^13^C NMR ^b,c^	No.	^1^H NMR ^a^	^13^C NMR ^b,c^
**2**	4.73, *bdd*, 7.8	64.4	**2a/b**	4.26, *bt*, 7.4	51.8
**3a/b**	3.53 (overlap) 3.14, *dd*, 2.6; 16.5	33.1	**3a/b**	3.25, *bt*, 5.0	28.0
**4**	7.01, *s*	113.6	**4**	7.21, *s*	115.7
**5**		143.0	**5**		147.7
**6**		146.0	**6**		149.8
**7**	6.84, *s*	99.0	**7**	7.15, *s*	101.2
**8**		137.5	**8**		138.4
**9**		123.2	**9**		127.3
**10**		176.9	**10d**		-
**11**	7.92, *bs* ^e^	142.0	**11**	8.41, *bs*, 12.2	146.3
**12**	5.71, *bs*	105.2	**12**	6.20, *bs*, 12.1	108.8
**13**		164.1	**13**		163.5
**14a/b**	2.80, *bd*, 2.70, *bd* ^e^	22.9	**14a/b**	3.04, *bt* ^e^, 7.6	24.8
**15a/b**	3.49 (overlap)	39.0	**15a/b**	3.63, *bt*, 8.2	40.7
**17**		157.9	**17**		151.4
**18**	6.19, *bs*	106.2	**18**	6.41, *bs*	106.2
**19 ^d^**		-	**19d**		-
**20**		165.2	**20**		165.7
**1′**	4.99, *d*, 7.5	101.2	**1′**	4.80, *d*, 7.4	104.1
**2′**	3.56 (overlap)	72.8	**2′**	3.49 (overlap)	77.4
**3′**	3.61 (overlap)	75.3	**3′**	3.51 (overlap)	74.2
**4′**	3.50 (overlap)	69.3	**4′**	3.42 (overlap)	71.2
**5′**	3.59 (overlap)	76.1	**5′**	3.44 (overlap)	78.3
**6′a/b**	3.91, *dd*, 1.5; 12.0 3.76, *dd*, 5.4; 12.4	60.3	**6′a/b**	3.92, *dd*, 1.7; 12.1 3.72, *dd*, 5.1; 12.4	62.4

^a 1^H NMR *δ* [ppm], mult, *J* [Hz]; ^b 1^H NMR *δ* [ppm]; ^c 13^C chemical shifts were derived from HSQC and HMBC; ^d^ The atom is not present; ^e^ *bs*, *bd,* or *bt*—broad singlet, broad doublet, or broad triplet, respectively.

## Data Availability

Not applicable.

## Supplementary Materials

The following supporting information can be downloaded at: https://www.mdpi.com/article/10.3390/ijms23031245/s1.
