# Supplementary material for: Dehydrogenation of Betacyanins in Heated Betalain-Rich Extracts of Red Beet (Beta vulgaris L.)
Source: Int J Mol Sci. 2022 Jan 23;23(3):1245. doi: 10.3390/ijms23031245 (PMC8835778; doi:10.3390/ijms23031245)
Supplement: Supplementary file 1 [file ijms-23-01245-s001.zip › ijms-1505046-supplementary.pdf]

Supplementary Materials

Dehydrogenation of Betacyanins in Heated Betalain-Rich Extracts of Red Beet (*Beta vulgaris* L.)

Katarzyna Sutor-Świeży [1], Michał Antonik [1], Justyna Proszek [1], Boris Nemzer [2,3], Zbigniew Pietrzkowski [4], Łukasz Popenda [5], Tomasz Świergosz [1] and Sławomir Wybraniec [1,*]

[1] Department C-1, Faculty of Chemical Engineering and Technology, Cracow University of Technology, ul. Warszawska 24, 31-155 Cracow, Poland; katarzyna.sutor@doktorant.pk.edu.pl (K.S.-Ś.); michal3antonik@gmail.com (M.A.); proszekjustyna@gmail.com (J.P.); tomasz.swiergosz@pk.edu.pl (T.Ś.)

[2] Research and Analytical Center, VDF FutureCeuticals, Inc., 2692 N. State Rt. 1-17, Momence, IL 60954, USA; bnemzer@futureceuticals.com

[3] Food Science & Human Nutrition, University of Illinois at Urbana-Champaign, 260 Bevier Hall, 905 S. Goodwin Ave., Urbana, IL 61801, USA

[4] Discovery Research Lab, VDF FutureCeuticals, Inc., 23 Peters Canyon Rd., Irvine, CA 92606, USA; zb@futureceuticals.com

[5] NanoBioMedical Centre, Adam Mickiewicz University Poznan, ul. Wszechnicy Piastowskiej 3, 61-614 Poznan, Poland; lukasz.popenda@amu.edu.pl

* Correspondence: slawomir.wybraniec@pk.edu.pl; Tel.: +48-12-628-3074; Fax: +48-12-628-2036

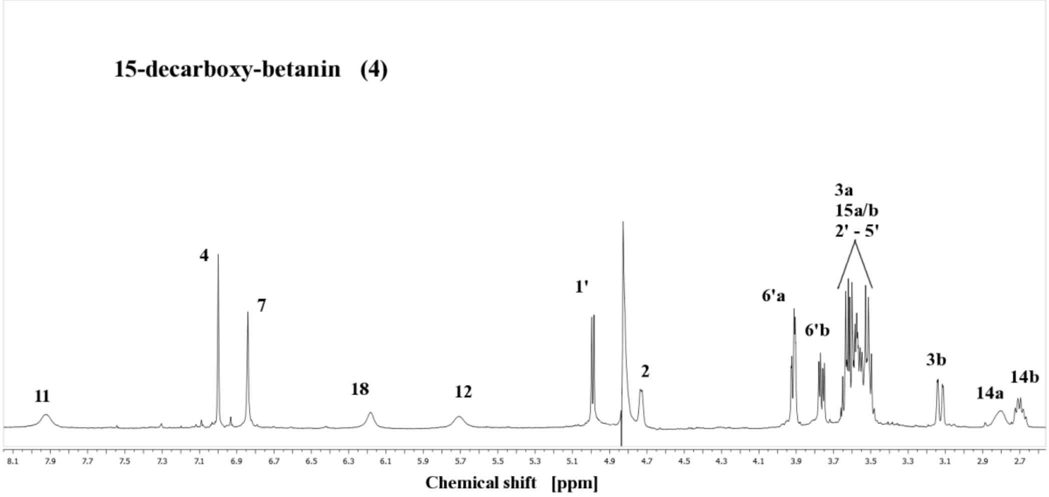

Figure S1. ^{1}H NMR spectrum of 15-decarboxy-betanin (D_2O, 298 K).

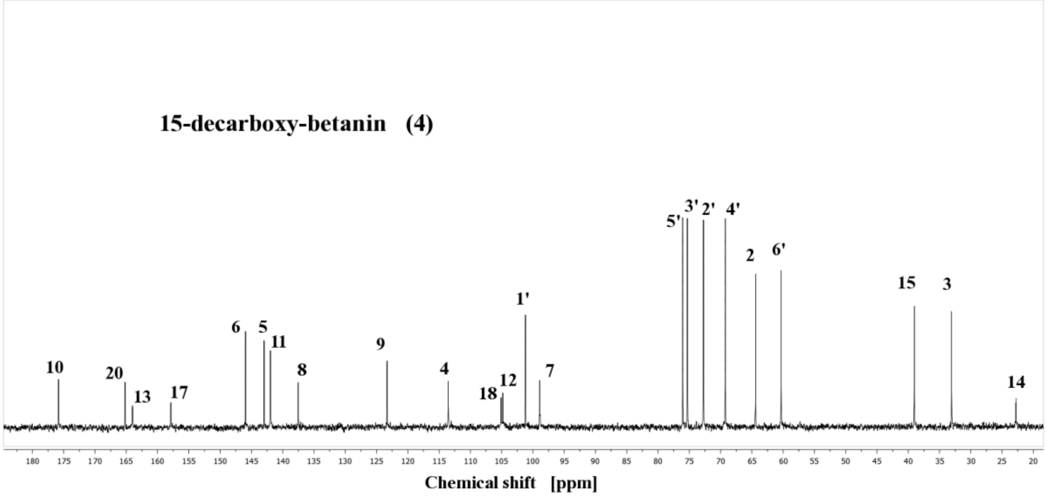

Figure S2. ^{13}C NMR spectrum of 15-decarboxy-betanin (D_2O, 298 K).

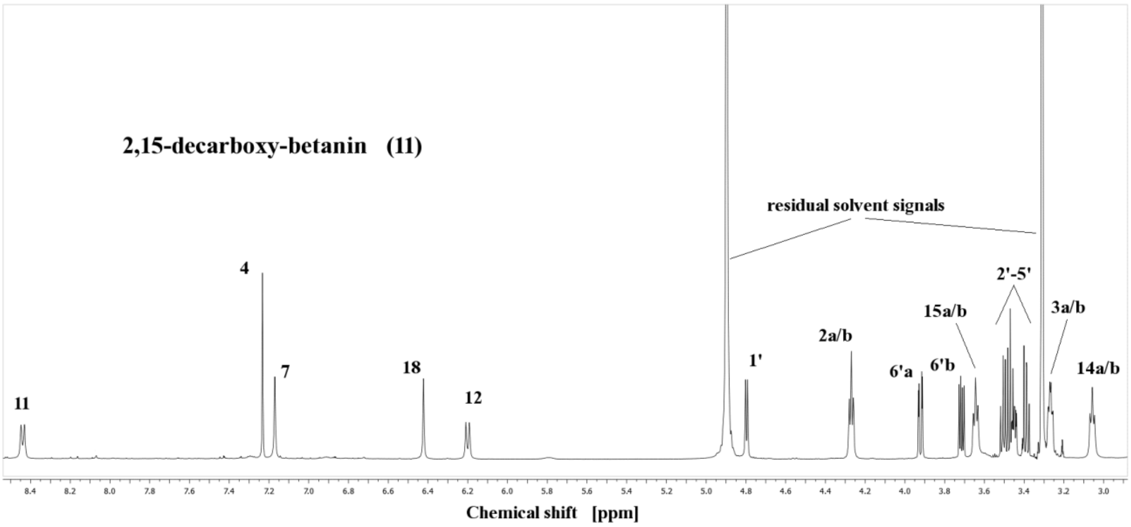

Figure S3. ^{1}H NMR spectrum of 2,15-decarboxy-betanin (CD_3OD, 295 K).

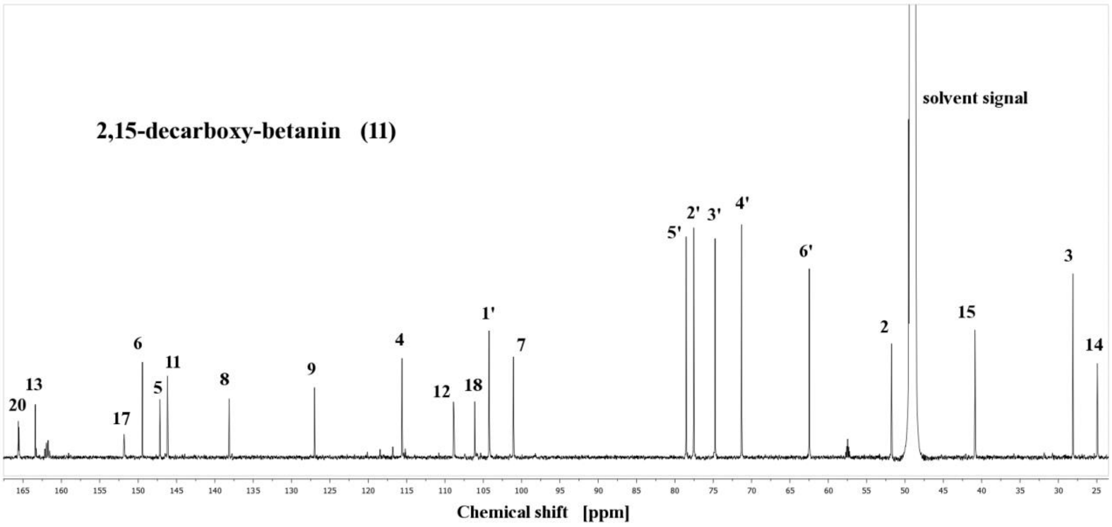

Figure S4. ^{13}C NMR spectrum of 2,15-decarboxy-betanin (CD_3OD, 295 K).
